# Preoperative chemotherapy and carbon ions therapy for treatment of resectable and borderline resectable pancreatic adenocarcinoma: a prospective, phase II, multicentre, single-arm study

**DOI:** 10.1186/s12885-019-6108-0

**Published:** 2019-09-14

**Authors:** Viviana Vitolo, Lorenzo Cobianchi, Silvia Brugnatelli, Amelia Barcellini, Andrea Peloso, Angelica Facoetti, Alessandro Vanoli, Sara Delfanti, Lorenzo Preda, Silvia Molinelli, Catherine Klersy, Piero Fossati, Roberto Orecchia, Francesca Valvo

**Affiliations:** 1 National Center of Oncological Hadrontherapy (Fondazione CNAO), Pavia, Italy; 2 General Surgery Department, Fondazione IRCCS Policlinico San Matteo, Pavia, Italy; 3 Department of Clinical, Surgical, Diagnostic and Pediatric Sciences, University of Pavia, Pavia, Italy; 4 Department of Oncology, Fondazione IRCCS Policlinico San Matteo, Pavia, Italy; 5 Hepatology and Transplantation Laboratory, Department of Surgery, Faculty of Medicine, University of Geneva, Geneva, Switzerland; 6 Divisions of Abdominal and Transplantation Surgery, Department of Surgery, Geneva University Hospitals, Geneva, Switzerland; 7 Anatomic Pathology Unit, Department of Molecular Medicine, University of Pavia and Fondazione IRCCS Policlinico San Matteo, Pavia, Italy; 8 Service of Clinical Epidemiology & Biometry, Fondazione IRCCS Policlinico San Matteo, Pavia, Italy; 9 MedAustron Ion Therapy Center, Wiener Neustadt, Austria; 10 European Institute of Oncology (IEO), Milan, Italy

**Keywords:** Pancreatic adenocarcinoma, Carbon ion radiation therapy, Chemotherapy, Surgery

## Abstract

**Background:**

Pancreatic adenocarcinoma is a high-mortality neoplasm with a documented 5-years-overall survival around 5%. In the last decades, a real breakthrough in the treatment of the disease has not been achieved. Here we propose a prospective, phase II, multicentre, single-arm study aiming to assess the efficacy and the feasibility of a therapeutic protocol combining chemotherapy, carbon ion therapy and surgery for resectable and borderline resectable pancreatic adenocarcinoma.

**Method:**

The purpose of this trial (PIOPPO Protocol) is to assess the efficacy and the feasibility of 3 cycles of FOLFIRINOX neoadjuvant chemotherapy followed by a short-course of carbon ion radiotherapy (CIRT) for resectable or borderline resectable pancreatic adenocarcinoma patients. Primary outcome of this study is the assessment of local progression free survival (L-PFS). The calculation of sample size is based on the analysis of the primary endpoint “progression free survival” according to Fleming’s Procedure.

**Discussion:**

Very preliminary results provide initial evidence of the feasibility of the combined chemotherapy and CIRT in the neoadjuvant setting for resectable or borderline resectable pancreatic cancer. Completion of the accrual and long term results are awaited to see if this combination of treatment is advisable and will provide the expected benefits.

**Trial registration:**

ClinicalTrials.gov Identifier: NCT03822936 registered on January 2019.

## Background

In the recent decades, pancreatic adenocarcinoma incidence has been increasing finally being the fourth biggest cause of cancer-related death in Europe with a 5-year-overall survival (OS) estimated around 5% [1].

Complete surgical resection is the only curative treatment, but unfortunately is available only up to 15–20% of all patients at the time of diagnosis. In the remaining patients, diagnosed in locally advanced stage (30–40%) with major vessel involvements either local tumor extension or systemic spread are obstacles for a surgical therapy [2]. When feasible complete surgical resection may lead to 5-years overall survival of 23.4%, but local control is not satisfying: hepatic metastases or local recurrence occur within 1–2 years from the surgery. Several studies have evaluated the role of neoadjuvant chemotherapy ± radiotherapy prompted by theoretical benefits in particular by the improvement of R0 resection rate and the analysis of the tumoral biological profile.

In the last years, several studies have evaluated the Gemcitabine combination (Gemcitabine + Cisplatin, Gemcitabine + Oxaliplatin, Gemcitabine + nab-Paclitaxel) vs Gemcitabine alone; patients treated with Gemcitabine combination seemed better than those treated with Gemcitabine monotherapy [3–6]. Neoadjuvant chemo-radiotherapy seems to be safe, with a low toxicity profile and low perioperative morbidity/mortality rate [7].

FOLFIRINOX has been shown to be superior to Gemcitabine alone in patients with advanced stages in terms of OS and response rate. Data on the efficacy of these regimes in resectable or borderline resectable pancreatic diseases are available [8, 9].

Tang et al. described a resectable rate of 80–100% in borderline resectable patients treated with a neoadjuvant approach [10]. The Japanese experience of carbon ion radiotherapy in treating locally advanced pancreatic disease appears to be effective and well tolerated. At the National Institute of Radiological Sciences (NIRS) in Chiba, Japan, several dose escalation studies have been conducted for the treatment of locally advanced pancreatic tumors with carbon ion therapy and concomitant Gemcitabine chemotherapy [11].

Subsequently, this experience has also been extended to resectable pancreatic adenocarcinoma. Although comparison of carbon ion radiotherapy with standard treatment in potentially resectable pancreatic cancer is difficult, NIRS results are promising in terms of resectability rate and over-all survival, if compared to treatment based on surgery alone or on chemotherapy, radiotherapy or chemoradiotherapy treatment combined with surgery. Preoperative Carbon Ion Radio-Therapy (CIRT) is expected to be effective in eliminating the retroperitoneal microinvasion of malignant cells, in reducing both the tumor size as well as the perivascular and the lymphatic involvement. Authors showed that a short course of neoadjuant CIRT (8 fractions of CIRT followed by surgery after 4 weeks) gave a high resectability rate in 21 out of 26 patients with a 5-year survival of 52% in operated patients and 42% in non-operated patients respectively [12–16].

From the radiobiological point of view, carbon ions features near the Bragg peak allow to deliver to the tumor a radiation that has a radiobiological efficacy comparable to the one of the neutron therapy, but with a lower Linear Energy Transfer (LET) in the entrance corridor and therefore producing less severe damage in healthy tissues. With the use of heavier ions like carbon, high-LET radiation effects translate into an increased relative biologic effectiveness (RBE) value by at least a factor of 2-fold or 3-fold relative to photons. Although RBE is an important factor, there are additional advantages associated with high-LET radiation that can contribute to survival benefits [17, 18]. In vitro and in vivo experimental studies reported the suppression of some pancreatic cells metastatic abilities, including migration and invasion by carbon ion treatment. At the same time, invasion and migration has been demonstrated to increase after photon radiation [19] Not less important, recent data described an increased immune-stimulatory effect after CIRT treatment compared to photons therapy [20].

Considering the suboptimal efficacy of conventional therapeutic alternatives and the consensus on the inclusion of patients with pancreatic cancer in clinical trials, these results give support for the administration of CIRT in resectable pancreatic tumors.

## Methods

### Study design and objectives

**Fig. 1:**
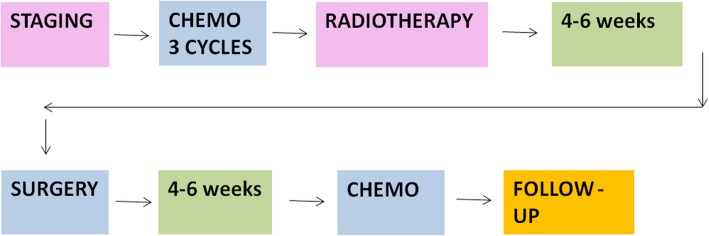
PIOPPO trial scheme

The proposed protocol is a prospective, phase II, multicentre, single-arm study. Thirty patients will undergo 3 cycles of FOLFIRINOX with restaging after the last cycle. Carbon ion radiotherapy will be planned with 4D imaging and will be delivered with respiratory gating and rescanning 4 times a week in 2 weeks. 4/6 weeks after carbon ion radiotherapy, after a restaging with a new abdomen CT scan, patients will undergo surgery. After 4/6 weeks, Gemcitabine will be administered for 6 cycles (Fig. 1).

The purpose of this trial is to assess the efficacy and the feasibility of the neoadjuvant administration of 3 cycles of FOLFIRINOX followed by a short-course of carbon ion radiotherapy (CIRT) for resectable or borderline resectable pancreatic adenocarcinoma patients before surgical resection.

The *primary outcome* of the study is the local progression free survival (L-PFS). L-PFS will be defined as the absence of locoregional failure.

The *secondary outcome* are:

- Overall survival (OS)
- Radical resectability rate (R0 resection) stratified for groups (resectable vs borderline resectable)

The R0 rate will be defined as the number of completed surgical procedures with histopathological confirmation of disease-free margins/number of enrolled patients. The resectability status is based on vascular involvement [21].

- Treatment toxicity (acute, intermediate, late)

Toxicity will be clinically evaluated according to CTCAE (Common Terminology Criteria for Adverse Events) scale version 4.0 [22] at least weekly during treatment, within 3 months from the CIRT (acute toxicity), from 3 to 6 months from the end of treatment (intermediate toxicity) and then during follow up visits beyond 6 months after CIRT completion (late toxicity).

- Intra and perioperative toxicity

In this trial Intra and perioperative toxicity will be scored with the Clavien-Dindo classification [23]:

- *Grade I*: Complications not requiring treatment
- *Grade II*: Complications requiring pharmacological treatment
- *Grade III*: Complications requiring surgical, endoscopic or radiological procedure without (IIIA) or with (IIIB) general anesthesia
- *Grade IV*: Complications that could be lethal requiring also intermediate care / intensive care unit including single (IVA) or multi- organ (IVB) dysfunction

Moreover quantitative surrogate endpoints will be recorded, i.e. operative time (in minutes), lost volume blood (in cc)

### Eligibility criteria

Patients meeting all of the following criteria will be considered for admission to the trial:

- Histologically/ cytologically confirmed pancreatic ductal adenocarcinoma
- Pancreatic adenocarcinoma defined as borderline resectable or resectable
- No distant metastases
- Age between 18 and 75 years
- Karnofsky performance status ≥70
- Absence of stomach and/or duodenum infiltration
- Ability of subject to understand character and individual consequences of the clinical trial
- Written informed consent prior to enrollment
- No critical complication or active double malignancy
- Adequate contraception when necessary
- Normal dihydropyrimidine dehydrogenase (DPD) enzyme activity
- Adequate hematopoietic function (neutrocytes, ≥1500/mm^3^; platelets, ≥10 × 10^4^/mm^3^ and hemoglobin, ≥9. g/dL), adequate hepatic function (total bilirubin ≤1.5 times institutional normal upper limit, albuminemia > 3 g/dL, serum creatinine ≤1.5 mg/dL)
- Ca19.9 ≥ 500 mg/dL and Bilirubine ≤1.5 times institutional normal upper limit are included (according to NCCN (version 2.2017) guidelines [24]

### Exclusion criteria

Patients meeting any of the following criteria will not be considered for the admission to the trial:

- Locally advanced non-resectable pancreatic cancer
- Neuroendocrine tumors
- Proof of distant metastases
- Low activity of DPD enzyme
- Compromised hepatic, renal and bone marrow function
- Documented neoplastic history with unfavorable prognosis
- Pregnancy status (verified by beta-HCG test)
- Breastfeeding status
- Presence of a definitive biliary metal stent
- Metal prothetic implant whose functions can be altered by high-energy radiation or which could compromise the target radiation region
- Documented contraindications to radiotherapy (exempli gratia: active infectious foci in irradiation area)
- Previous radiation treatment or implantation of abdominal radioactive seed
- Patients declared unfit for surgery
- Patients with a history of mental illness
- Patients who can not comprehend the purpose of the procedure or who are unable to sign the written consent form

### Design and study procedures

PIOPPO trial is a prospective, phase II, multicentre, single-arm study.

### Chemotherapy

Enrolled patients with a resectable or borderline resectable pancreatic cancer will undergo the scheme FOLFIRINOX (Oxaliplatin 85 mg / sq. m g1 + Irinotecan 180 mg / sq. m g1 + calcium levofolinato 200 mg / sq. m g1 + 5-fluorouracil 400 mg / sq. m bolo g1 + 5-fluorouracil 2400 mg / sq. m infusion 48 h continuous g1 q14) for 3 cycles, followed by disease reassessment.

### Carbon ion radiotherapy

All patients will be positioned in customized cushions and immobilized with a solid thermoplastic mask. A tight mask, fitted on the patient abdomen at end expiration and rapidly cooled, will be used to achieve mild uniform abdominal compression. Typically two immobilizations will be performed: one in prone and one in supine position. A set of 2-mm-thick 4D computed tomography (CT) images will be taken for treatment planning in each position. The Anzai system (Anzai Medical, Tokyo, Japan) will be used to acquire the patient breathing signal for retrospective 4D CT reconstruction [25, 26]. Four respiratory phases are reconstructed: end inspiration, end expiration, 30% of the surrogate marker signal dynamic before end expiration, and 30% of the surrogate marker signal dynamic after end expiration.

The tumor extent will be evaluated by CT and, when necessary, fluorodeoxyglucose positron emission tomography (FDG PET).

The radiation oncologists will define the clinical target volume (CTV) as the gross tumor volume (GTV) with a 5-mm margin and the locoregional elective lymph node and neuroplexus region. The locoregional elective lymph node region includes the celiac, superior mesenteric, peripancreatic, portal, and para-aortic region for pancreatic head cancers and the splenic region for pancreatic body and tail cancers according to Japan Pancreas Society classification (i.e. stations number 8,13,14,16,17 for tumors of the head and stations 8,9,11,14,16,18 for tumors of the body tail) [27].

The planning target volume (PTV) includes the clinical target volume with a 5-mm margin to account for set up uncertainties and residual tumor motion. CIRT treatment plans will be optimized with the Syngo RT Planning (Siemens Medical Systems, Germany) Treatment Planning System on the CT scan corresponding to the maximum expiration phase of each 4D CT acquisition.

Typically three beam directions will be used: antero-posterior in supine position, postero-anterior in prone position and right-left through the liver. Each day two beams will be simultaneously applied. In single cases it will be acceptable to have only one beam in one of the two positions. When necessary plans are adapted with help structures and specific constraints to increase robustness. Every day the patient will be treated either in prone or in supine position. Treatment is performed combining gating and rescanning with a ≈ 1 s gating window centered around the maximum expiration phase. Plan robustness against residual motion is evaluated recalculating dose distribution of optimized particle fluence on the + − 30% phases (treated as static images) as representative of the gating window boundaries.

Interplay effect is considered negligible because of the reduced respiratory motion (thanks to abdominal compression) 5 times rescanning and number of fractions greater than 10. Inter-fraction variability of respiratory motion and organ filling is accounted for with a minimum of 2 re-evaluation 4DCT scans: one before treatment start and one after the first 4 fractions.

Full details of the organ motion coping strategy will be reported in a separate paper.

Doses of carbon ion are expressed in photon equivalent doses, defined as the physical doses multiplied by the relative biologic effectiveness of the carbon ions. Patients will receive CIRT at the dose of 38.4 Gy [RBE] carried out in 8 fractions, 4 fractions per week.

The dose constraints will be:

- Spinal cord: D_max_ 30 Gy [RBE]
- Stomach and small bowel: D_max_ 38 Gy [RBE], D5cc < 36 Gy [RBE]
- Liver: V18Gy < 700 cc
- Kidney: D_mean_ < 15 Gy [RBE]

### Surgery

Before surgery, restaging CT scans will be performed to evaluate resectability and absence of systemic progression. 4 to 6 weeks after the completion of CIRT, patients will undergo surgical resection as follows:

- pancreaticoduodenectomy for tumors of the pancreatic head
- distal pancreatectomy and splenectomy for tumors of the body or tail

### Pathological findings

The following characteristics of the tumor will be recorded:

- size
- presence/absence of vascular and/or perineural invasion
- state of surgical margins
- tumor involvement of pancreatic surface
- state of regional lymph nodes
- grade of regression scored according to Evans et al. scheme [28]

### Sequential chemotherapy

In the post-operative period, adjuvant chemotherapy is administered from 30 to 40 days after surgery, according to the Gemcitabine Scheme 1000 mg / m2 g1, 8, 15 q28 for 6 cycles, as expected in clinical practice.

### Statistical methods and sample size

The calculation of sample size for PIOPPO trial is based on the analysis of the primary endpoint “progression free survival” according to Fleming’s Procedure. According to literature data [29, 30], the expected probability of success at 24 months (H_0_: *p* < =0.35) is 35%, considering 60% the desirable probability of success (H_1_: *p* > 0.35). Therefore, with 26 patients we will be able to reject the null hypothesis with a alfa-error of 0.038 and a power of 80%. Considering a dropout rate of 13%, 30 patients will be enrolled in the study. The null hypothesis will be rejected if the number of “responder” is ≥14.

All enrolled patients will be evaluated for the efficacy endpoints (ITT population). Patients who underwent the treatment will be evaluated for the safety endpoints (safety population).

Subjects meeting the enrolment criteria who will decline to participate, and thus will not be enrolled, will serve as concurrent controls.

Continuous variables will be reported as number, mean, standard deviation, median, interquartile range, minimum and maximum value. Categorical variable will be described as nominal value and %.

The survival curves will be estimated using the Kaplan Meier method: the cumulative probability of PFS will be calculated and a 95% confidence intervals will be given.

Follow-up time will be calculated from the signature of the informed consent to the first date of progression or death. If 14 or more patients have reached the primary endpoint of the study, treatment will be declared more effective than the historical, and worthy of further comparative phase II / III studies. For descriptive purposes, the cumulative incidence of progression will also be calculated considering death as a competitive risk. The cumulative incidences of progression or death will be graphically illustrated.

#### Recruitment period, follow- up duration and location ratio

The overall duration of the treatment from the enrolment to the surgery, is expected to be 14 weeks. The recruitment period will last 5 years consisting in 3 years of enrollment and 2 years of follow-up.

Patients will be followed by CT, MRI or PET scans every 3 to 6 months. Local recurrence will be defined in terms of lesions occurring in the planning target volume based on CT, MRI, or PET scans. The absence of local recurrence will be described as local control.

#### Ethics, informed consent and safety

The treatment protocol for the current study was reviewed and approved by Pavia Ethical Committee at Fondazione IRCCS Policlinico San Matteo, (number: 20180033297 dated March, 14th, 2018). All patients signed the informed consent form before the initiation of therapy.

#### Data handling, storage and archiving of date

All findings including clinical, radiological and laboratory data will be documented by the investigator or an authorized member of the study team in the subject’s medical record and in the eCRF. Investigators are responsible for ensuring that all sections of the eCRF are completed correctly and that entries can be verified against source data. Investigators guarantee the privacy of patients and personal data are treated according to the Italian Law (D.Lgs. 10 agosto 2018, n. 101) and the European General Data Protection Regulation (EU 2016/679). The data will be stored at least 10 years. All data obtained for this study will be entered into a local regulation compliant Data Management System provided by the Fondazione IRCCS Policlinico San Matteo, Pavia. The RedCap platform, resident on a secure server at the Fondazione, will be used for that purpose. All users will be identified through an individual username and password. All data entry, modification or deletion will be recorded automatically in an electronic audit trail.

Data reported in the eCRFs should be consistent with and substantiated by the subject’s medical record and original source documents. The eCRF data will be monitored by the Coordinating Center or designee. The final, completed eCRF Casebook for each subject must be electronically signed and dated by the PI within the EDC system to signify that the Investigator has reviewed the eCRF and certifies it to be complete and accurate. The Sponsor will retain the final eCRF data and audit trail. A copy of all completed eCRFs will be provided to the investigator.

## Discussion and conclusion

Pancreatic cancer behaves as a systemic disease and any effort must be multimodal and should include both systemic chemotherapy, radiation therapy and surgery. The traditional approach to the resectable pancreatic cancer is surgery followed by adjuvant chemotherapy or chemoradiation therapy. However, the benefits of a neodjuvant chemoradiation has been showed in several studies and in medical practice. Pancreatic cancer is an aggressive disease with poor survival also in localized resectable cases. This manuscript describes an Italian, prospective, phase II, multicentre, single-arm study designed to assess the efficacy and the feasibility of 3 cycles of FOLFIRINOX neoadjuvant chemotherapy followed by a short-course of CIRT for resectable or borderline resectable pancreatic adenocarcinoma patients.

The trial was opened to accrual in January 2018 and more Institutions are going to be involved to further increase the accrual of patients. Since January 2018 six patients have been so far enrolled and five have completed the surgical phase. There has been no dropout. Despite an initial slow accrual the enrollment has accelerated in the last 4 months and it is expected that the study will be completed, if not on time, with minimal delay. Very preliminary results provide initial evidence of the feasibility of the combined chemotherapy and CIRT in the neoadjuvant setting for resectable or borderline resectable pancreatic cancer. Moreover, CIRT does not affect negatively the surgical approach. Completion of the accrual and long term results are awaited to see if this combination of treatment is advisable and will provide the expected benefits.

If the trial will pass its phase 2, the authors will investigate the possibility to open a phase 3 trial that will be based also on the results of the other studies currently in progress given the clinical relevance of the topic*.*

## Data Availability

Data sharing is not applicable to this article as no datasets were generated or analysed during the current study.

## Abbreviations

4D: Four Dimension
CIRT: Carbon ion radiotherapy
CNS: Central Nervous System
CT: Computed Tomography
CTCAE: Common Terminology Criteria for Adverse Events
CTV: Clinical Target Volume
D_max_: Maximum Dose
Dmean: Mean Dose
DPD: Dihydropyrimidine Dehydrogenase
eCFR: Electronic Case Report Forms
FDG PET: Fluorodeoxyglucose Positron Emission Tomography
GTV: Gross Tumor Volume
ITT: Intention To Treat
LET: Linear Energy Transfer
L-PFS: Local Progression Free Survival
MRI: Magnetic Resonance Imaging
NCCN: National Comprehensive Cancer Network
NIRS: National Institute of Radiological Sciences
OS: Overall Survival
PTV: Planning Target Volume
RBE: Relative Biologic Effectiveness
RT: Radiation Therapy
SMA: Superior Mesenteric Artery
β-HCG: Beta Human Chorionic Gonadotropin
